# COVID-19 related stigma and health-protective behaviours among adolescents in the Netherlands: An explorative study

**DOI:** 10.1371/journal.pone.0253342

**Published:** 2021-06-22

**Authors:** L. C. Preusting, M. P. Raadsen, A. Abourashed, H. A. C. M. Voeten, M. N. Wagener, E. de Wit, E. C. M. van Gorp, L. Doornekamp

**Affiliations:** 1 Department of Viroscience, Erasmus MC, University Medical Center Rotterdam, Rotterdam, The Netherlands; 2 Faculty of Science, VU University, Amsterdam, The Netherlands; 3 Municipal Public Health Service Rotterdam-Rijnmond, Rotterdam, The Netherlands; 4 Department of Public Health, Erasmus MC, University Medical Center Rotterdam, The Netherlands; 5 Center of Expertise Innovations in Care, Rotterdam University of Applied Science, Rotterdam, The Netherlands; 6 Athena Institute for Research on Communication and Innovation in Life and Health Sciences, VU University, Amsterdam, The Netherlands; 7 Department of Internal Medicine/Infectious Diseases, Erasmus MC, University Medical Center Rotterdam, Rotterdam, The Netherlands; Middlesex University, UNITED KINGDOM

## Abstract

The COVID-19 pandemic has forced many governments to impose social distancing measures upon its citizens, including in the Netherlands. Motivating adolescents to adhere to measures such as social distancing can be challenging, since adolescents are relatively more affected by them, while experiencing virtually no personal health benefit. In addition, the COVID-19 pandemic seems to impact the social environment of adolescents in schools, as some media sources have reported bullying and stigmatisation of students with an Asian appearance. This study aims to explore the experiences of adolescents regarding their Health-Protective Behaviour (HPB), as well as the prevalence and expression of stigma towards ethnic minorities within the context of the first wave of COVID-19 pandemic. We performed a cross-sectional mixed-methods study, including two independent online questionnaires. An adapted version of the “HPB” questionnaire (n = 528) and the “Measure of Disease-Related Stigma (MDRS)” questionnaire (n = 380), were administered to Dutch adolescents of 10–16 years old, attending primary or secondary school. Furthermore, 15 interviews were held with eight male and seven female adolescents. All data collection took place between March 17 and April 20, 2020. Results show that adolescents perceive COVID-19 as a threat to other peoples’ health, rather than their own, and report adherence to public health measures in the interest of older and more vulnerable members of their community. We found no convincing evidence for widespread misinformation or stigmatising of certain ethnic groups among adolescents related to COVID-19 during this study. Participants acknowledged such behaviour happened in the early stages of the pandemic, before this study was initiated. Adolescents are a vital group for public health researchers to engage with during a pandemic, even when reaching them can be challenging.

## Introduction

The COVID-19 pandemic, caused by the Severe Acute Respiratory Syndrome coronavirus 2 (SARS-CoV-2), is an ongoing serious public health threat. Unprecedented actions have been taken to limit the spread of the virus, with many governments worldwide placing restrictions on their populations’ freedom of movement and implementing compulsory personal hygiene and social distancing measures. Compliance with such measures cannot be ensured using enforcement alone, and therefore, monitoring the motivation of the population to adhere to recommendations is a crucial part of the public health response to this crisis [1]. Behavioural and social sciences can inform public health communication strategies aimed at improving a population’s ability to limit the spread of COVID-19 [2].

Previous research particularly focused on Health-Protective Behaviour (HPB) of adults during outbreaks of emerging infectious diseases, especially during the H1N1 influenza pandemic in 2009 and the SARS outbreak in 2003 [1, 3–5]. HPB, defined as “*any behaviour performed by a person*, *regardless of his or her perceived or actual health status*, *in order to protect*, *promote or maintain his or her health*, *whether or not such behaviour is objectively effective towards that end*” [6] plays a significant role in the prevention of further spread of a virus and control of an outbreak [1, 3]. A very suitable theoretical framework to study HPB in context of an infectious disease outbreak is the Protection Motivation Theory (PMT), as it involves how people respond to a threat [7]. This framework served as a theoretical background in HPB research during SARS and Influenza outbreaks [3, 5] and could therefore be useful to study HPB during COVID-19 pandemic as well. The PMT indicates that motivation to engage in HPB is influenced by four factors: perceived severity of the threat and personal vulnerability (threat appraisal); perceived efficacy of the recommended behaviour and self-efficacy (coping appraisal) [7]. Self-efficacy is defined as: *“the belief that one is capable of performing the recommended behaviour”* [8].

Adolescents, being defined as individuals in the 10–19 years age group [9], present a unique challenge in regard of HPB, due to their relative autonomy compared to younger children and still developing cognitive ability to cope with risk and responsibility. Their low personal risk of severe COVID-19 [10–12] may provide little intrinsic incentive to adhere to protective measures. Instead, their compliance with public health guidelines must be motivated by a sense of obligation to others. Questions as to whether adolescents play a role in SARS-CoV-2 transmission or if they could be expected to comply to social distancing measures have raised doubts whether these measures should be implemented for this age group at all.

Social environments, such as classrooms and online (social media) platforms, are places for peer-interaction, which can foster positive attitudes and behaviours but can also be places for bullying and stigmatisation. Historically, large scale infectious disease outbreaks have been associated with social unrest and stigmatisation of minority groups that are blamed for such disasters [13, 14]. Similarly, incidents of stigmatisation and bullying of people of Asian descent were reported in the Netherlands in the early stages of the COVID-19 outbreak [15, 16].

Social stigma with reference to an outbreak is the perception that a particular person or group of persons that share certain characteristics are linked to a disease and are therefore blamed, labelled, discriminated or stereotyped [17]. Social stigma is an important aspect of infectious disease outbreaks [14, 18], but it is not covered in the PMT model. An appropriate model to study disease-related stigma is the Attribution Theory of Stigma (ATS) [18, 19]. The ATS consists of four ordered factors: the perceived controllability of the event; the perceived responsibility; an emotional reaction (either sympathy or anger); and the behavioural reaction (either helping or rejecting) [19]. During an outbreak of an emerging infectious disease, public health agencies have to maintain a balance between protecting the public and refrain from methods that could inadvertently lead to stigma [14]. Research has shown that individuals who perceive themselves at risk of the disease are likely to engage in protective behaviour but, at the same time, may also stigmatise people who are believed to be potential sources of the disease [1]. Additional research shows that persons who are feared and stigmatised during an outbreak show a delay in seeking detection and treatment, which complicates the control of an outbreak [13, 14, 20]. Finally, as was observed during the Ebola epidemic, survivors of the disease may be confronted with stigma, rejection, and blame for spreading the disease [21].

The issues described above, instigated this study, focused on exploring the relations between the components of the PMT among adolescents and to explore the existence of stigma towards certain subpopulations within the context of the first wave of the COVID-19 pandemic during which schools were closed in the Netherlands. At that given time, adolescents in the Netherlands were dealing measures such as online education, keeping 1.5 meters distance from others, staying away from elderly and vulnerable people and staying home as much as possible. This is one of the first studies that focused on HPB and stigma among adolescents during a pandemic, a group which comprises about one-sixth of the population in the Netherlands [22]. As such, the aim of this research is twofold: to contribute to a better understanding of the HPB of adolescents to COVID-19 and to explore the presence of stigma among adolescents from both primary and secondary schools in the Netherlands during the COVID-19 pandemic.

## Materials and methods

This is a cross-sectional study, using a mixed-methods approach. Semi-structured interviews were combined with two questionnaires to integrate in-depth qualitative information with large-volume quantitative information.

### Participants and procedures

The target population of this research were Dutch adolescents between the ages of 10 and 16 years old. Therefore, participants were either in the final two years of primary school or in the first three years of secondary school. In the Netherlands, there are different levels of secondary education: preparatory vocational secondary education, senior general secondary education, and university preparatory education (in Dutch ‘VMBO’, ‘HAVO’, and ‘VWO’ respectively), in order of academic aptitude [23]. Adolescents of all levels participated in this study. Data collection took place during the period in which schools were closed in the Netherlands due to the COVID-19 pandemic. The questionnaires were administered between March 17 and April 20, 2020. All interviews were held between April 2 and April 16, 2020.

### Sampling strategies

Selected schools were a convenience sample drawn from a pool of schools that had previously participated in Viruskenner, an educational programme on prevention of virus infections [24]. For the questionnaires, schools were approached by email and asked for their cooperation in recruiting students. Of the 20 schools that were approached to contribute to this research, one primary school and five secondary schools were willing to participate. All adolescents aged 10–16 years old attending the participating schools were sent links via email to both our self-administered online questionnaires on the web-based platform Qualtrics®. The questionnaires were provided separately to minimize potential dropout rate and maximize the proportion of students finishing at least one of the two questionnaires.

Participants for the interviews did not participate in the questionnaires and were selected by convenience sampling based on relationships with the researchers and stratified by educational level and type of school. Adolescents were selected until data saturation was reached. Due to social isolation measurements during the time of this study, interviews were performed via telephone calls.

### Data collection

#### Questionnaires

The quantitative part of this research was performed by using two questionnaires based on the PMT and ATS frameworks [7, 19]. The questionnaire to explore HPB, based on the PMT [7], was adapted from an existing validated questionnaire used during the first SARS outbreak [5]. A few questions were altered or deleted to make the questionnaire suitable for adolescents, and, also, some questions were added based on other questionnaires that were developed by the same researchers [25].

The questionnaire to explore disease-related stigma, based on the ATS [19], was adapted from the Measure of Disease-Related Stigma (MDRS) scale [26]. Stump and colleagues developed this scale, validated it, and tested it in the contexts of three different diseases of which one was a human immunodeficiency virus (HIV) infection. The MDRS scale was designed around a vignette to prime the participants for a certain situation. Three different vignettes have been used, namely: a high onset-controllability condition, a low onset-controllability condition, and an unknown onset-controllability condition. For this study, the vignettes were adapted to the context of the COVID-19 pandemic. In short, in the high onset-controllability condition, an adolescent from China infected with SARS-CoV-2, who is aware of the outbreak in her country, moved to the Netherlands. In the low onset-controllability condition, a Dutch adolescent travelled to China and returned to the Netherlands infected with SARS-CoV-2 but was not aware of the COVID-19 outbreak in China. The unknown onset-controllability condition describes a Dutch adolescent infected with SARS-CoV-2 who does not know how she got infected with the virus. Thus, the difference between the vignettes is solely focused on the controllability part. At the start of the questionnaire, participants were randomly assigned to one of these three vignettes. The “MDRS” questionnaire was translated from English to Dutch, with minor adjustments. The questionnaire consisted of 26 five-point Likert-type scale items.

Both questionnaires were piloted with two adolescents of the lowest age categories of this study, to confirm whether the questions were understandable for adolescents of this age.

#### Interviews

The qualitative part of this research consisted of semi-structured interviews. A topic list for the interviews was designed in advance together with the co-authors, based on the components of the PMT and ATS frameworks [7, 19]. The interviews were performed by the first author via telephone call with the participants.

### Data analysis

#### Quantitative analysis

The questionnaires were retrieved from the Qualtrics server and only the ones that were 100 percent completed were included in the analysis (see S1–S3 Appendix). For the “HPB” questionnaire, descriptive analyses were performed for the perceived severity, vulnerability, response efficacy, self-efficacy and sources of information. Due to the large sample size (n = 528), normality of the data was assumed, and parametric tests were performed [27]. Paired-sample t-tests were performed to indicate the difference between the means. Spearman’s rank correlations were calculated on the relation between perceived severity, vulnerability, response efficacy, and self-efficacy. A multivariate regression analysis of variance was performed to measure the effect size of the participants baseline characteristics on perceived severity, perceived vulnerability, response efficacy, and self-efficacy. If a participant chose the option “I don’t know”, this was reported as a missing value. A preliminary factor analysis was conducted on all 26 five-point Likert-type scale items from the MDRS scale. A final factor analysis was conducted on the 22 items with oblique rotation. The Kaiser-Meyer-Olkin (KMO) measures verified the sampling adequacy for the analysis. Finally, four Likert scales were computed, which were taken to be continuous interval scales [27]. Means were calculated for the four different scales in each of the three vignette groups and independent sample t-tests were performed to indicate the significance of the different means. Pearson correlations coefficients were calculated between the four Likert scales and the controllability vignette being either low or high. In all analyses, performed with IBM SPSS version 24, p<0.05 was considered significant.

#### Qualitative analysis

Interviews were transcribed ad verbatim and analysed using the systematic procedures of the three-step coding method: deductive coding, axial coding and selective coding [28] based on constructs of the PMT and ATS.

### Ethical considerations

The study was carried out in accordance with the Declaration of Helsinki. In this study, participants were not subjected to procedures nor were required to follow rules of behaviour. Therefore, according to Dutch law, this study was exempt from institutional review board approval requirements [29].

Written informed consent for contacting students was obtained first from school administrators and subsequently from parents or legal guardians, giving them the opportunity to opt-out on behalf of their child. Additionally, written informed consent was obtained from the students themselves upon starting the online questionnaire. Questionnaires were performed anonymously. Before the interviews, verbal informed consent was obtained from all participants and we subsequently asked for permission to record audio of the interview. Recordings were solely used for transcription and transcripts were coded.

## Results

### Quantitative results

#### Participants and characteristics

The “HPB” and “MDRS” questionnaires were initiated by 748 and 650 adolescents and finished by 539 and 383 participants respectively. After excluding participants outside of the predefined age range (10–16 years), being mostly teachers and parents examining our material, 528 and 380 questionnaires remained for analysis respectively.

Table 1 presents the baseline characteristics of the participants of both the “HPB” questionnaire and the “MDRS” questionnaire. Approximately 40 percent of the participants were male. About 10 percent of the participants attend primary school. The largest part of the participants attends “VWO” (university preparatory education). The proportion of participants that had an autochthonous Dutch background, was approximately 70%, with the remainder reporting a foreign background, originating from a wide variety in countries in all continents except Oceania and Antarctica.

**Table 1:** Baseline characteristics of the participants.

		Questionnaire HPB *n* = 528	Questionnaire MDRS *n* = 380
Gender: *n* (%)	Male	210 (39.8)	145 (38.2)
	Female	311 (58.9)	232 (61.1)
	Unknown	7 (1.3)	3 (0.8)
Age: mean±SD		13.1±1.27	13.0±1.28
Education year: *n* (%)	Primary school group 7	18 (3.4)	12 (3.2)
	Primary school group 8	32 (6.1)	31 (8.2)
	Secondary school 1^st^ grade	170 (32.2)	118 (31.1)
	Secondary school 2^nd^ grade	161 (30.5)	123 (32.4)
	Secondary school 3^rd^ grade	147 (27.8)	96 (25.3)
School level: *n* (%)	Primary school	50 (9.5)	43 (11.3)
	Secondary school VMBO*	30 (5.7)	12 (3.2)
	Secondary school VMBO/HAVO*	17 (3.2)	21 (5.5)
	Secondary school HAVO*	78 (14.8)	53 (13.9)
	Secondary school HAVO/VWO*	91 (17.2)	108 (28.4)
	Secondary school VWO/Gymnasium*	262 (49.6)	143 (37.6)
Ethnicity: *n* (%)	Autochthonous Dutch	378 (71.6)	259 (68.2)
	Other	150 (28.4)	121 (31.8)
Participation in Viruskenner: *n* (%)	Yes	111 (21)	82 (21.6)
	No	417 (79)	298 (78.4)

*Note*: *There is overlap in participants between the two questionnaires since many participants administered both questionnaires*.

*VMBO = preparatory vocational secondary education; HAVO = senior general secondary education; VWO/Gymnasium = university preparatory education.

#### HPB: Knowledge

The first three questions of the questionnaire tested basic knowledge about COVID-19. Almost all participants (99.8%) were aware that SARS-CoV-2 is contagious, 92% agreed that some people infected with SARS-CoV-2 do not get ill and 96% knew that people with COVID-19 could die from the infection. Overall, 88% answered all three questions correctly and 11% made one mistake. A weak but statistically significant correlation was found between the test score (1 point per correct answer) and age (r_s_ = 0.154, p<0.01) or school grade (r_s_ = 0.143, p<0.01).

#### HPB: Perceived threat

**Fig 1:**
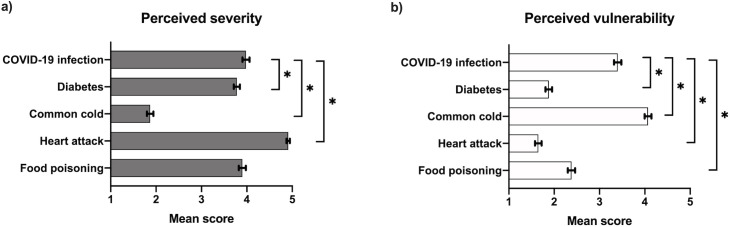
Mean scores of perceived severity and perceived vulnerability (scale 1–5) regarding a COVID-19 infection, common cold, diabetes, heart attack and food poisoning. *Note*: (a) Perceived severity question: How serious would you rate the following diseases? (b) Perceived vulnerability question: What are the odds that you will get these diseases within a year? Scores: 1 = not at all serious/very unlikely, 2 = not serious/unlikely, 3 = not unserious and not serious/not likely and not unlikely, 4 = serious/likely, 5 = very serious/very likely. Error bars describe 95% confidence intervals. *significance between means for a coronavirus infection compared to the four diseases, p<0.05, two-tailed.

Perceived severity, perceived vulnerability, fear, and concern of COVID-19 infection, compared to common infectious and non-infectious diseases, were measured on a five-point Likert-type scale (1 being least serious or equivalents, 5 being most serious or equivalents). The mean perceived severity of COVID-19 reported by participants (3.98 [95% CI: 3.90–4.06]) was significantly higher than their perceived vulnerability (3.4 [95% CI: 3.32–3.48]) (Fig 1). Participants rated fear and concern for COVID-19 on average 2.66 [95% CI: 2.58–2.74] and 3.17 [95% CI: 3.30–3.24] respectively, which were both significantly lower than their perceived severity.

The mean scores of perceived severity and vulnerability for a COVID-19 infection were compared to four other diseases. The mean perceived severity for a COVID-19 infection was significantly higher than a common cold (1.87 [95% CI: 1.79–1.94]) and diabetes (3.78 [95% CI: 3.71–3.85]), but lower than a heart attack (4.91 [95% CI: 4.87–4.95]) (Fig 1A). Furthermore, the mean perceived vulnerability for a COVID-19 infection was significantly higher than diabetes (1.88 [95% CI: 1.81–1.95]), food poisoning (2.38 [95% CI: 2.30–2.46]), and a heart attack but significantly lower than a common cold (4.07 [95% CI: 4.00–4.14]) (Fig 1B).

#### HPB: Perceived response- and self-efficacy

**Fig 2:**
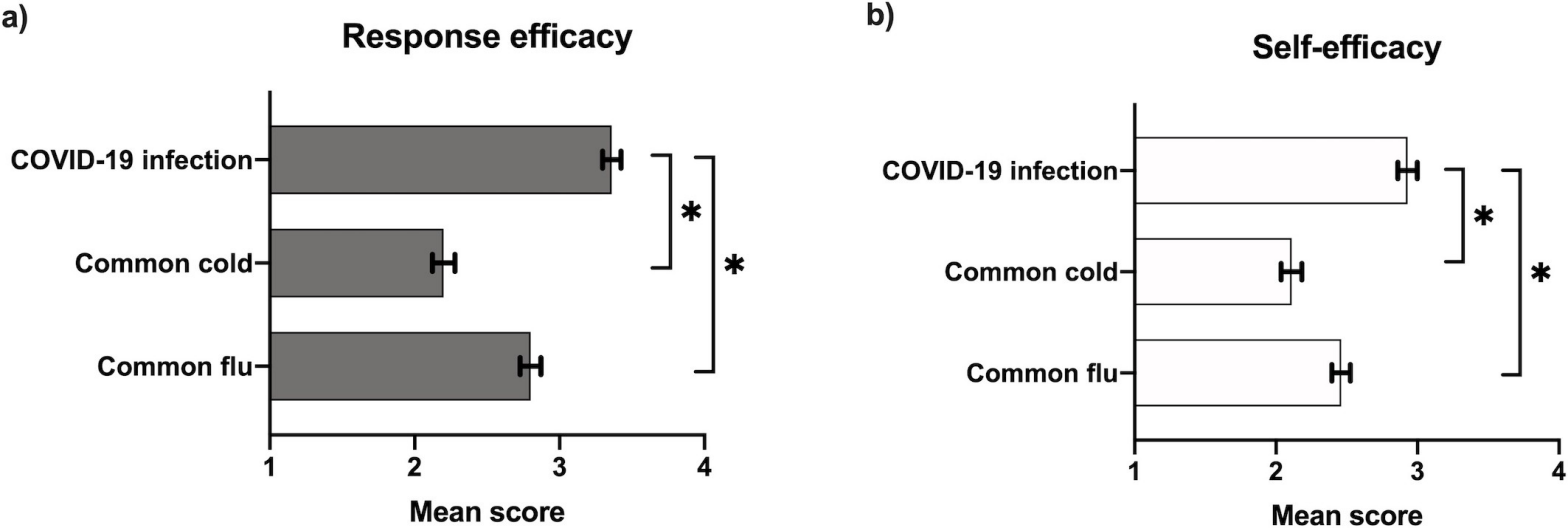
Mean scores of response efficacy and self-efficacy (scale 1–4) regarding common flu, coronavirus and common cold. *Note*: (a) Response efficacy question: Do you think it will help to take precautionary measures to protect yourself from “*disease*”? (b) Self-efficacy question: Do you think you could protect yourself from “*disease*”? Scores: 1 = not at all, 2 = a little bit, 3 = quite a bit, 4 = definitely. Error bars describe 95% confidence intervals. *Significance between means for a coronavirus infection compared to the two other diseases, p<0.01, two-tailed.

Participants rated the response efficacy of measures to prevent COVID-19 significantly higher (3.36 on a four-point Likert-type scale [95% CI: 3.30–3.42]) than self-efficacy (2.93 [95% CI: 2.86–3.00]) for COVID-19 (Fig 2A). Both the response efficacy and self-efficacy were rated significantly higher for a COVID-19 infection than for the flu (2.80 [95% CI: 2.73–2.87], 2.46 [95% CI: 2.40–2.52]) or a common cold (2.20 [95% CI: 2.12–2.28], 2.11 [95% CI: 2.04–2.18]) (Fig 2B).

**Fig 3:**
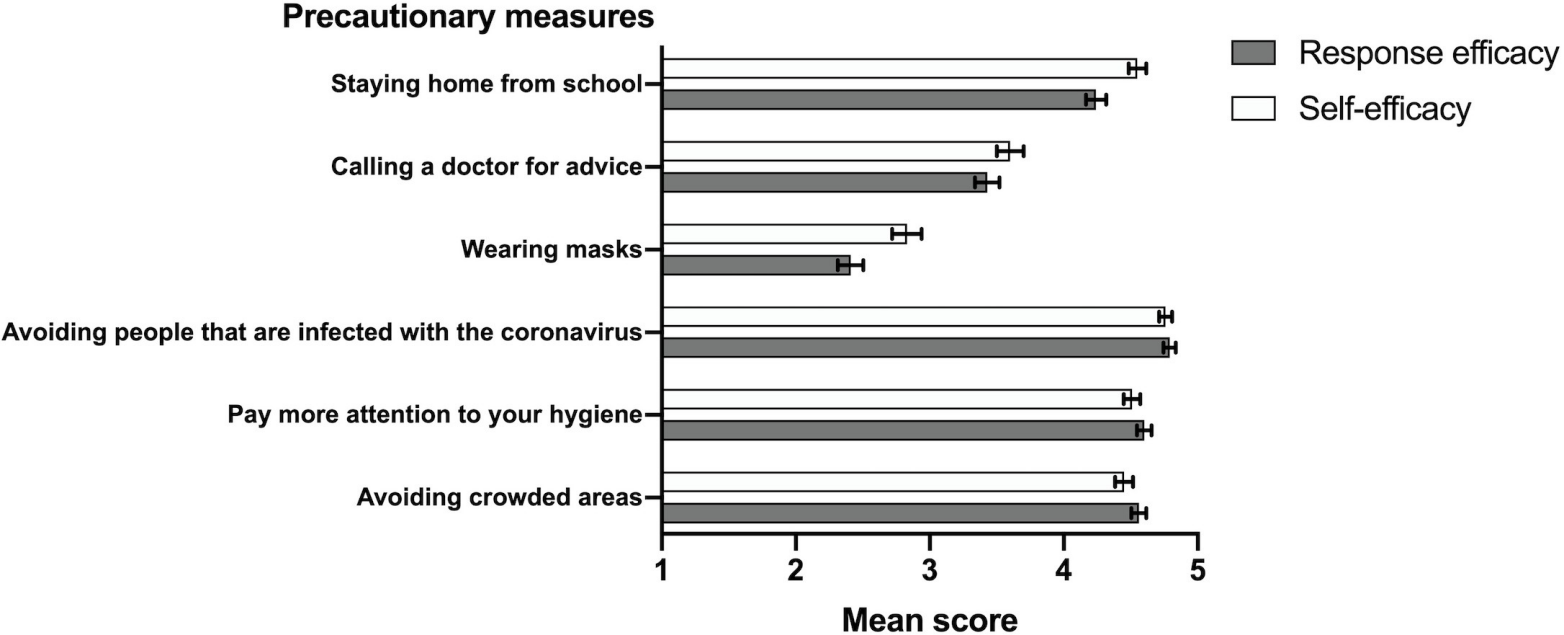
Mean scores of response efficacy and self-efficacy (scale 1–5) regarding five different precautionary measures to prevent COVID-19. *Note*: Response efficacy question: Of the following precautionary measures, do you think they will help in preventing a coronavirus infection? Self-efficacy question: If the government would advise one of these following precautionary measures, would you be capable to adhere to them? Scores: 1 = certainly not, 2 = probably not, 3 = not probable and not improbable, 4 = probably, 5 = certainly. Error bars describe 95% confidence intervals.

The mean scores of response- and self-efficacy for individual precautionary measures to prevent COVID-19 infection were compared, showing a strong consistency between response and self-efficacy and high scores for avoiding crowded areas, paying more attention to hygiene, and avoiding people that are infected with the coronavirus (Fig 3). Mean scores of both response- and self-efficacy for wearing masks were significantly lower. Adolescents rated themselves as capable of staying home and wearing masks, more than they rated these measures to be efficient.

#### HPB: Correlations between components of the PMT

Table 2 shows the Spearman correlation coefficients between four different components. Response- and self-efficacy rates were positively correlated (r_s_ = 0.371, p<0.05). Furthermore, perceived severity was significantly positively correlated with both response efficacy (r_s_ = 0.242, p<0.05) and self-efficacy (r_s_ = 0.211, p<0.05). Also, perceived vulnerability was negatively correlated with self-efficacy (r_s_ = -0.213, p<0.05).

**Table 2:** Spearman correlations (R_s_) between fear, concern, perceived severity, vulnerability, response efficacy and self-efficacy.

	Perceived severity	Perceived vulnerability	Response efficacy	Self-efficacy
Perceived severity	1			
Perceived vulnerability	-.072	1		
Response efficacy	.242*	-.023	1	
Self-efficacy	.211*	-.213*	.371*	1

*Correlation is significant at p<0.05 (two-tailed).

#### HPB: Sources of information

Use and reliability of several sources of information were measured on a five-point Likert-type scale (1 being not at all (reliable), 5 being very much (reliable)). Overall, the most used sources of information to obtain information about COVID-19 were television, family, internet, and social media (mean scores 3.94, 3.81, 3.29 and 3.16 respectively). The television (4.19) and family (3.69) were also both perceived as reliable. However, the internet (3.05) and social media (2.55) were perceived as less reliable. Furthermore, the newspaper was not often used as a source of information (1.95) but was perceived as a reliable source (4.06).

#### HPB: Multivariate regression analysis of variance

There was no significant effect of gender, age, ethnicity, or school level on the perceived severity, perceived vulnerability, response- and self-efficacy. Participation in the Viruskenner program had a significant but very weak effect on self-efficacy (Λ = 0.97, *F*(4,410) = 3.21, p = 0.07, partial η^2^ = 0.017).

#### Stigma: Factor and reliability analysis

Of the 26 five-point Likert-type scale items from the MDRS scale, four items did not cluster with their dimension according to the MDRS in the factor analysis and were excluded from further analysis. Based on 22 five-point Likert-type scale items, the sampling adequacy for the analysis was ‘marvellous’ (KMO = 0.917) [30]. Five factors had ‘eigenvalues’ over the Kaiser’s criterion of 1 and in combination explained 70% of the variance. The items that clustered on the same factor suggest that the first factor represents cognitive attributions (11-item-scale), the second factor anger (3-item-scale), the third sympathy (3-item-scale), the fourth social distancing (2-item-scale) and the fifth helping (3-item-scale). The reliability analysis showed that all scales had high reliabilities except for the social distancing scale (Cronbach’s α = 0.909; 0.935; 0.704; 0.478 and 0.895 respectively).

#### Stigma: Descriptive statistics

Mean scores on five-point Likert-type scale items for cognitive attributions and anger were higher in the high controllability group (2.56 and 2.34) than in the low controllability group (2.20 and 1.86) (1 being strongly disagree, 5 being strongly agree). Conversely, the means for sympathy and helping were higher in the low controllability group (3.92 and 3.88) than the high controllability group (3.72 and 3.68). In all vignettes, sympathy and helping determinants were rated significantly higher than the anger and cognitive attributions determinants (p<0.01).

#### Stigma: Correlations between controllability level, cognitive attributions, sympathy, and helping behaviour related to COVID-19

Table 3 shows Pearson correlation coefficients for the controllability level, and for the cognitive attributions, anger, sympathy, and helping behaviour scales. Positive associations were found between the controllability level, cognitive attributions and anger emotions and between sympathy and helping behaviour. Cognitive attributions and anger were most strongly correlated (r = 0.572, p<0.01). Both cognitive attributions and anger were negatively and correlated with sympathy and helping.

**Table 3:** Pearson correlations (r) for the four reliable scales of MDRS and the controllability level.

	Controllability level	Cognitive attributions	Anger	Sympathy	Helping behaviour
Controllability level	1				
Cognitive attributions	.240*	1			
Anger	.234*	.572*	1		
Sympathy	-.117	-.253*	-.324*	1	
Helping behaviour	-.093	-.175*	-.184*	.351*	1

*Correlation is significant at p<0.05 (two-tailed).

### Qualitative results

#### Characteristics of the interview participants

Three interviewees were primary school children and the remaining twelve interviewees were secondary school children. All interviewees were children living in the Netherlands, of which two had Turkish background and three had Asian background (see S4 Appendix).

#### HPB: Sources of information

The effect of the closure of schools in the Netherlands was described as a reason for having to rely more on media and family to gain information on the pandemic, rather than from the teachers at school. Adolescents were critical of some of the sources of information they used. For example, one interviewee talked about conspiracy theories that exist on the internet and indicated that not all information from this source should be trusted. Another interviewee stated that he searched for information on the online social media platform Reddit but reflected with scepticism on what he found, saying: *“I don’t know if everything I know is true*, *because I obtained some of the information from the internet and then there is a chance that it is just not true*. *I also use the social media page Reddit a lot*, *on which everyone can post something*, *but whether that is really reliable*?*”* (R11). All interviewees acknowledged the national press-conferences by the government as an important reliable source of information.

Although all respondents indicated that they thought knowledge about COVID-19 was important, they expressed various motivations. Ten interviewees were motivated by the social impact of the outbreak. A male student mentioned: *“Everybody is involved in the coronavirus situation*, *so I think it is important to be aware of the coronavirus and to watch the news”* (R12). Five interviewees stated that they were looking for guidance on how to modify their behaviour, with one student articulating: *“I think it is important because then I know if I can go outside or not and how much distance I need to keep from others and stuff like that”* (R3). Three interviewees said that they were interested in the virus itself. A female student mentioned: *“I think this subject is very interesting and therefore I looked it up a lot on the internet”* (R1).

#### HPB: Perceived threat

When asked about the severity of COVID-19, all interviewees acknowledged that they could get COVID-19, but most of them did not think that they would get seriously ill. Only one interviewee had a higher perceived severity for getting infected herself, she mentioned: *“I think it is dangerous because I’ve heard that it can damage your lungs”* (R1). Most interviewees perceived COVID-19 as a higher threat to other people then to themselves, as one female student mentioned: *“I think it is very dangerous for other people such as elderly people and people with a weak immune system”* (R9). They mainly regarded it as a threat to older individuals or people with underlying health conditions and viewed compliance to precautionary measures as a way of protecting them.

Besides the perceived severity and vulnerability, emotions were also identified as a theme that influenced the adolescents’ perceived threat. Unlike in the questionnaires, in which fear was an infrequently reported emotion, in the interviews, adolescents expressed fear that people close to them would get sick. A female student stated: “*I think my parents might catch it or anyone I know; I am very afraid of that and I think about it a lot”* (R2). Others expressed fear as a result of the unusual and powerful nature of the pandemic. Uncertainty about the future and how long the pandemic would continue was another reason for anxiety and frustration. Adolescents expressed grief as a result of empathy with those who had lost their lives or good health to COVID-19 and their relatives.

#### HPB: Compliance with precautionary measures related to costs and rewards

All participants indicated that they perceived the recommended behavioural guidelines by the government as important to reduce the spread of COVID-19. They all took precautionary measures such as hand washing and physical distancing to others and avoiding contact with the elderly or those with poor health. Missing out on leisure activities, social contact, and sportsmanship were mentioned as factors complicating compliance to these measures. Only one interviewee mentioned the economic damage to his family and others due to the lockdown but did not believe it outweighed its necessity. The rewards mentioned for acting upon the recommended behaviour were almost exclusively intrinsic. A few interviewees reported experiencing some extrinsic benefits from the precautionary measures, related to the comfort of being able to attend classes online.

Despite these obstacles, similarly to respondents to the questionnaires, interviewees generally reported high self-efficacy and response efficacy. Maintaining physical distance from others while outdoors had become routine for some, requiring little conscious effort.

#### Stigma: Causal controllability

According to all interviewees, the most plausible origin of the COVID-19 outbreak was zoonotic. Most considered human behaviour to have contributed to the emergence of the virus. One male interviewee said: *“I hear that someone ate a bat and that’s the reason that the coronavirus is in the human world”* (R13). One-third of the interviewees stated that the pandemic could have been prevented, mostly through public health actions. A male student mentioned: *“They want to forbid the animal market in China; if they had done that earlier then the outbreak would not have happened”* (R3). Two-thirds of the interviewees argued that the COVID-19 outbreak could not have been prevented because this outcome could not have been anticipated. 12 out of 15 interviewees believed there was initial secrecy by Chinese authorities and that this contributed to early warning signs being ignored and delayed implementation of control measures. However, only a few students believed that, and even if this had not been the case, the pandemic could have been prevented.

The most mentioned perceived cause of the spread of COVID-19 from China to other countries was the travel behaviour of humans, as one male interviewee said: *“The disease is very contagious and many people travel*, *so it spread very fast”* (R3). All interviewees stated that the spread of the virus to other countries was not preventable, mainly due to a lack of knowledge at the early stages of the outbreak.

On the other hand, multiple interviewees argued that the spread could have been restricted. For instance, a female student mentioned: *“Maybe countries could have closed their borders earlier to reduce the spread of the coronavirus*, *or maybe countries could have acknowledged the virus as a more serious problem*, *because people noticed too late that they should take precautions and then the virus had already spread everywhere”* (R1).

Interviewees regarded unsafe behaviour and poor compliance to health guidelines as important contributors to the spread of COVID-19 in the Netherlands. According to all interviewees, COVID-19 is controllable but not preventable. For example, a female student mentioned: *“If everyone just sticks to the rules*, *then nobody gets infected anymore … Some people just don’t adhere to the rules”* (R9).

#### Stigma: Perceived responsibility

None of the interviewees made any direct reference to the responsibility of specific countries for spreading COVID-19. They did note inconsistencies between the COVID-19 policies between countries, as a male student said: *“I think it’s strange that not all countries do have similar rules for the coronavirus”* (R11).

When introducing China as possible origin of the outbreak, two-thirds of interviewees expressed that they do not hold the country responsible for the start of the pandemic. The remaining ones did not express strongly held opinions, except one who believed the Chinese people were responsible due to aspects of their culinary practices.

Eight of the interviewees made statements about personal responsibility of individuals for contracting and spreading COVID-19. Of them, only one interviewee convincingly held others responsible for spreading the virus and their infection, as she said: *“People should have taken it more seriously and taken more measures to prevent it from spreading as we do”* (R2). The other seven interviewees doubted but tended more towards not holding them responsible. An example of an answer that a male student gave was: *“There were people who were infected with the coronavirus who still travelled*, *but on the other hand these people could not have known that they had the virus I think”* (R11). No specific groups or ethnicities were mentioned by adolescents as being more to blame than others.

#### Stigma: Emotional reactions

From all interviews, sympathetic and neutral emotions towards people infected with COVID-19 were found most frequently. In parallel with the findings from the questionnaires, subjects attributing greater responsibility towards COVID-19 infected individuals, also experienced more antipathy towards them. Interviewees who mentioned inconsistencies in policies between countries experienced more antipathetic emotions towards this state of affairs, though not directed against any specific country or people. Antipathy was expressed as disappointment or general frustration, but never as anger or hatred. When confronted with media reports on discrimination against people of Asian descent related to COVID-19, all interviewees strongly objected to this behaviour. Two interviewees reported having observed discrimination within this context in the early phase of the pandemic but not anymore at the time of interviewing.

## Discussion

### Principal findings

In this study, we explored HPB of and stigma among adolescents during the early COVID-19 pandemic. Adolescents in this study reported that they perceived COVID-19 as a threat to another individuals’ health, rather than their own and adhered to public health measures in the interest of older members of their community. We found no convincing evidence of widely held misinformed beliefs, conspiracy theories or prejudices against certain ethnic groups among adolescents related to COVID-19 at the research period of this study (March 17 until April 20, 2020). When asked explicitly about such tendencies as reported by national media at the time, interviewees indicated that this was an early reaction to the pandemic, which had been resolved by the time this study was initiated.

### Context of this study

The expressed risk perceptions of participants and their concerns for others may be explained by the fact that youths are considerably less susceptible to severe COVID-19 infection, which justifies this belief [10–12, 31]. However, the Dutch Institute for Public Health and the Environment (RIVM) has recently conducted a survey among adults with an average age of 65 in the Netherlands and found that individuals in this age group also regard COVID-19 as a severe risk for others rather than just themselves [32]. While, in contrast to youths, the risk of severe disease and death from COVID-19 in this demographic group is not negligible, even among individuals with no significant comorbidities [31]. This begs the question whether both adolescents and adults are affected by optimism bias [33] when assessing their personal health risk from COVID-19 or only the adults, with adolescents simply making a rational judgement. This bias was observed previously in the Netherlands during the first SARS outbreak among adults [3] and more specifically among Dutch adolescents in health issues to which they do have a higher risk, such as sexually transmitted diseases [34]. Furthermore, optimism bias was also observed among adolescents in the United States (US) [35], indicating that adolescents, in general, could be susceptible to optimism bias. An Australian study investigated perceptions about severity and vulnerability in adults during the COVID-19 pandemic as well. In contrast to the findings of the Dutch RIVM, Australian adults perceived themselves highly vulnerable to SARS-CoV-2 and were highly concerned for themselves [36]. This study may indicate that optimism bias does not appear everywhere and might be culture-bound, as also reported in other research [37].

Interviewed adolescents mentioned to adhere to most of the recommended precautions because they were motivated to protect the elderly and vulnerable people from COVID-19. In line with these findings, other—mainly European—research among adults on the motivation to adhere to distancing measures also demonstrated that empathy for the most vulnerable to COVID-19, promotes adhering to the recommended precautionary measures [38]. The same effect is found for being subjected to prosocial messages that induce thoughts on the potential to infect vulnerable individuals [39]. Contradictory, research in the US showed that messaging on recommended behaviour during the COVID-19 pandemic using a self-focused frame was equally effective to a close prosocial frame [40]. Therefore, the impact of prosocial messaging may depend on the degree of prosociality in a country [41].

Supporting a more rational risk perception among adolescents is the fact that their responses to questionnaires and interviews are generally consistent with official and verified sources of information [42]. Despite adolescents in 2020 being arguably more exposed to social media and potential misinformation compared to older generations during their adolescence, they appear to be very capable in assessing the reliability of information they receive. We found no evidence of widely held misinformed beliefs, conspiracy theories or prejudices. Our questionnaires showed that, during the peak of the COVID-19 outbreak in the Netherlands, stigma regarding people with Asian appearance was not strongly present. This finding was confirmed in the interviews, which revealed that the stigma was present at the time of the media reports in the early phase of the pandemic, but was extinguished at the time of interviewing. Also, we found positive relations between the perceived controllability, responsibility and anger emotions, which were all negatively related to sympathy and helping behaviour. These correlations were also observed in earlier research using the same questionnaire in an HIV setting [26] and were in line with the relations the ATS suggests [19]. Similar relations were also observed during our interviews. Altogether, this may indicate that these correlations show how stigma could have risen in the early stages of the COVID-19 pandemic.

Interestingly, the questionnaire results showed a higher response efficacy among adolescents compared to self-efficacy, indicating they appreciate the necessity of the precautionary measures that have been implemented in the Netherlands but found adhering to all of them demanding. Some interviewees broke the rules on social distancing, leading to rewards as social contact, sportsmanship, and pleasure, possibly to counteract the response costs. These response costs, and rewards earned by not engaging in protective behaviour, could explain the lower self-efficacy mean. This explanation is in line with the literature reporting that missing out on social activities and sports strongly contributes to this [43]. Precautionary measures such as staying home from school, avoiding contact with other children, and not being able to go to sports anymore have a high impact on children and could lead to learning difficulties and loneliness [43, 44].

We found correlations between the perceived severity of COVID-19 and response- and self-efficacy, which would all lead to a motivation to protect oneself according to the PMT [7]. Our interviews confirmed that participants who perceived COVID-19 as severe or thought that the recommended behaviour by the government was important, also engaged in protective behaviour, indicating a positive relation between the severity, response efficacy and behaviour. These correlations were also seen in previous research. For example, during the avian influenza outbreak between 2006–2007 in the Netherlands, it was demonstrated that the perceived severity, vulnerability, and self-efficacy were significantly related to precautionary behaviour [45]. Also, during the first SARS outbreak, response- and self-efficacy were related to precautionary behaviour [4]. Unexpectedly, we found a negative correlation between the perceived vulnerability and self-efficacy, for which we have no explanation and we propose that this relation could be investigated in further research.

### Implications for future research

The qualitative results suggest that two components might strengthen the PMT [20], which should be confirmed and further investigated in future research. Firstly, emotions may be added as a component of the threat appraisal, because interviewees frequently expressed their emotions such as anxiety, frustration, and fear to indicate they perceived COVID-19 as a threat. The link between risk perception and emotions was also found in COVID-19 health seeking-behaviour among adults [46], indicating the importance of studying the role of emotions as part of the PMT. Secondly, it could be argued that the benefits of engaging in protective behaviour, which may be called ‘response benefits’, should be considered as part of the coping appraisal, as interviewees also experienced some extrinsic benefits from the precautionary measures. Future experimental research to determine the effects of exposure to these PMT components on HPB could be of particular interest. Furthermore, future research efforts could provide more insights on changes in HPB and stigma over time by using a longitudinal design.

### Implications for public health communication

Adolescents in this study were aware that knowledge of COVID-19 was crucial for them to perform HPB in line with the recommendations. In the absence of clear and unambiguous sources of reliable information, adolescents are potentially vulnerable to misinformation spread on social media, which are frequently used as a primary source of information in this demographic [47, 48]. During a pandemic, politicians should focus on communicating reliable information and information sources to adolescents and stimulate teachers to spread correct information even in times of closed schools. Adolescents communicated to adhere to the recommended precautions by the government if ‘response benefits’ outweigh the possible response costs of engaging in protective behaviour. Therefore, public health agencies should focus on communicating the urgency and importance of the precautionary measures and highlight the ‘response benefits’ specific for adolescents. Furthermore, adolescents indicated their motivation to adhere to recommended precautions in the interest of the vulnerable. Therefore, sending prosocial messages that induces thoughts of infecting vulnerable individuals could the promote effects of empathy and HPB among Dutch adolescents [38, 39].

During the research period of this study, we found no prejudices against certain ethnic groups among adolescents related to COVID-19. Qualitative results showed that stigma was observed early in the outbreak of COVID-19, at time during which there were many uncertainties about the virus. We showed high awareness concerning COVID-19 among adolescents participating in this study, indicating that stigma could have disappeared because the general public developed a better understanding of the virus. The notion that an accurate understanding of a pandemic, including its cause, possible treatments, and prevention strategies, helps in preventing stigma was also reported in previous research [14]. Therefore, we suggest that to counteract disease-related stigma that could arise in future pandemics, public health agencies should perform actions focussing on promoting an accurate understanding of the outbreak, directly from the start of an outbreak.

### Strengths and limitations

Some limitations have to be considered when interpreting the results of this study. Firstly, the study was performed in a convenience sample of schools which bears the risk of selection bias. The study was conducted during the peak of the initial COVID-19 outbreak in the Netherlands and only a minority of the schools that were approached were able or willing to accommodate it (six out of 20, mostly higher educational level schools). This limited our sample size and may have skewed the selection of schools towards students with a more affluent socioeconomic background. This has undoubtedly reduced the power of this study to detect rare, but potentially relevant cases of stigma and discrimination. Nevertheless, the study population includes adolescents from all education levels and was spread over four regions in the Netherlands, ensuring a broad representation in terms of academic aptitude and geography. Another limitation is that the “HPB” questionnaire lacked questions about motivation and adhering to protective behaviour. Therefore, relations between the components of the threat and coping appraisals of the PMT and adolescents’ actual protective behaviour could not be calculated from the questionnaire data. Counteracting this limitation, in our qualitative part of this research, interviewees were asked on all coping and threat components as well as their motivation and actual behaviour, to get an impression of the connections between all components. Lastly, a limitation to this study was that this research has a cross-sectional design and therefore has not captured behaviour of youths earlier in the COVID-19 outbreak, which is when media reports on discrimination of Dutch-Asian individuals first appeared, neither after the peak of the outbreak. On the other hand, this research was performed during the peak of the COVID-19 outbreak in the Netherlands when the topic was on top of the participants’ minds, which strengthens our results. During the interviews, the respondents’ experience of the start of the outbreak was discussed, which was only a few weeks before the timing of the interview, minimizing recall bias. The questionnaires used for this study were previously validated, but were slightly altered to make them more suitable for the target demographic group, and questions from other questionnaires were also included. Although this could have affected their reliability and reproducibility, factor and internal consistency analysis showed robust results.

### Conclusion

Based on this research we conclude that Dutch adolescents performed HPB during the COVID-19 pandemic mainly to protect others. Stigma arose early in the COVID-19 pandemic, but extinguished during the peak of the outbreak in the Netherlands according to interviewed adolescents. Circumstances during a public health crisis of this magnitude can be volatile and may well cause drastic and sudden shifts in motivation of and intention to health-behaviours of youth. Detecting such changes early could be used to adapt health communication strategies by public health organisations, educational institutions and healthcare providers.

## Supporting information

S1 Appendix Dataset health-protective behaviour questionnaire. (XLSX)

S2 Appendix Dataset measure of disease-related stigma questionnaire. (XLSX)

S3 Appendix Legends questionnaire datasets. (DOCX)

S4 Appendix Demographics of interview participants. (DOCX)

## References

[1] Brug J, Aro AR, Oenema A, De Zwart O, Richardus JH, Bishop GD. SARS risk perception, knowledge, precautions, and information sources, the Netherlands. Emerg Infect Dis. 2004 Aug; 10(8): 1486–1488. doi: 10.3201/eid1008.040283 15496256 PMC3320399

[2] Van Bavel JJ, Baicker K, Boggio PS, Capraro V, Cichocka A, Cikara M, et al. Using social and behavioural science to support COVID-19 pandemic response. Nat Hum Behav. 2020; 4(5):460–471 doi: 10.1038/s41562-020-0884-z 32355299

[3] De Zwart O, Veldhuijzen IK, Elam G, Aro AR, Abraham T, Bishop GD, et al. Perceived threat, risk perception, and efficacy beliefs related to SARS and other (emerging) infectious diseases: results of an international survey. Int. J. Behav. Med. 2009 Jan 6; 16(1): 30–40. doi: 10.1007/s12529-008-9008-2 19125335 PMC2691522

[4] Jiang X, Elam G, Yuen C, Voeten H, De Zwart O, Veldhuijzen I, et al. The perceived threat of SARS and its impact on precautionary actions and adverse consequences: a qualitative study among Chinese communities in the United Kingdom and the Netherlands. Int. J. Behav. Med. 2009 March 10; 16(1):58–67. doi: 10.1007/s12529-008-9005-5 19277874 PMC7090686

[5] Voeten HA, de Zwart O, Veldhuijzen IK, Yuen C, Jiang X, Elam G, et al. Sources of information and health beliefs related to SARS and avian influenza among Chinese communities in the United Kingdom and The Netherlands, compared to the general population in these countries. Int. J. Behav. Med. 2009 Jan 29; 16(1):49–57. doi: 10.1007/s12529-008-9006-4 19184453 PMC7090907

[6] Harris DM, Guten S. Health-protective behavior: An exploratory study. J Health Soc Behav. 1979 March; 20(1):17–29. 438490

[7] Maddux JE, Rogers RW. Protection motivation and self-efficacy: A revised theory of fear appeals and attitude change. J Exp Soc Psychol. 1982 Aug 3; 19(5):469–479.

[8] Conner M, Norman P. Predicting Health Behaviour. 2^nd^ ed. England: Open Press University; 2005

[9] Adolescent health [Internet]. WHO; 2020 [Cited 2020 July 12]. Available from: https://www.who.int/southeastasia/health-topics/adolescent-health

[10] Lee PI, Hu YL, Chen PY, Huang YC, Hsueh PR. Are children less susceptible to COVID-19? J Microbiol Immunol Infect. 2020 Feb 25; 53(3):371–372. doi: 10.1016/j.jmii.2020.02.011 32147409 PMC7102573

[11] Ludvigsson JF. Systematic review of COVID‐19 in children shows milder cases and a better prognosis than adults. Acta Paediatr. 2020 Mar 23; 109(6):1088–1095. doi: 10.1111/apa.15270 32202343 PMC7228328

[12] Qiu H, Wu J, Hong L, Luo Y, Song Q, Chen D. Clinical and epidemiological features of 36 children with coronavirus disease 2019 (COVID-19) in Zhejiang, China: an observational cohort study. Lancet Infect Dis. 2020 Mar 25; 20(6):689–696. doi: 10.1016/S1473-3099(20)30198-5 32220650 PMC7158906

[13] Barret R, Brown P. Stigma in time of influenza: social and institutional responses to pandemic emergences. JID. 2008 Feb 15; 197(1):34–37. doi: 10.1086/524144 18269326

[14] Person B, Sy F, Holton K, Govert B, Liang A. Fear and stigma: the epidemic within the SARS outbreak. Emerg Infect Dis. 2004 Feb; 10(2):358–363. doi: 10.3201/eid1002.030750 15030713 PMC3322940

[15] Hofland T. Voorzitter Chinese studentenvereniging: discriminatie is sinds corona next level. Erasmus magazine [internet]. 2020 Feb 11 [Cited 2020 July 10]; Available from: https://www.erasmusmagazine.nl/2020/02/11/voorzitter-chinese-studentenvereniging-discriminatie-is-sinds-corona-next-level/

[16] Rademaker G. ’Stinkchinees!’ Dit is wat Chinese Nederlanders naar hun hoofd geslingerd krijgen sinds het uitbreken van het coronavirus. Eenvandaag avrotros [internet]. 2020 Feb 13 [Cited 2020 July 10]; Available from: https://eenvandaag.avrotros.nl/panels/opiniepanel/alle-uitslagen/item/stinkchinees-dit-is-wat-chinese-nederlands-naar-hun-hoofd-geslingerd-krijgen-sinds-het-uitbreken/

[17] Social stigma associated with COVID-19. [Internet]. WHO; 2020 [Cited 2020 July 20]. Available from: https://www.who.int/docs/default-source/coronaviruse/covid19-stigma-guide.pdf?sfvrsn=226180f4_2

[18] Mak WW, Mo PK, Cheung RY, Woo J, Cheung F, Lee D. Comparative stigma of HIV-AIDS, SARS, and Tuberculosis in Hong Kong. Soc Sci Med. 2006; 63(7):1912–1922. doi: 10.1016/j.socscimed.2006.04.016 16766106 PMC7115765

[19] Weiner B, Perry RP, Magnusson J. An attributional analysis of reactions to stigmas. J Pers Soc Psychol. 1988; 55(5):738–748. doi: 10.1037//0022-3514.55.5.738 2974883

[20] Chesney M A, Smith AW. Critical delays in HIV testing and care: The potential role of stigma. Am Behav Sci. 1999 Apr; 42(7):1162–1174.

[21] Karamouzian M, Hategekimana C. Ebola treatment and prevention are not the only battles: understanding Ebola-related fear and stigma. Int J Health Policy Manag. 2015 Jan; 4(1):55. doi: 10.15171/ijhpm.2014.128 25584356 PMC4289040

[22] Bevolking; geslacht, leeftijd en burgerlijke staat, 1 januari. [Internet]. CBS; 2019 Dec 2 [Cited 2020 July 20]. Available from: https://opendata.cbs.nl/statline/?dl=1EFBB#/CBS/nl/dataset/7461bev/table

[23] Education in the Netherlands. [Internet]. Nuffic; 2020 [Cited 2020 July 11]. Available from: https://www.nuffic.nl/en/subjects/education-in-the-netherlands/

[24] Doornekamp L, Stegers-Jager KM, Vlek OM, Klop T, Goeijenbier M, van Gorp EC. Experience with a multinational, secondary school education module with a focus on prevention of virus infections. Am J Trop Med Hyg. 2017 Jul 12; 97(1):97–108. doi: 10.4269/ajtmh.16-0661 28719318 PMC5508890

[25] Bults M, Beaujean DJ, de Zwart O, Kok G, van Empelen P, van Steenbergen JE, et al. Perceived risk, anxiety, and behavioural responses of the general public during the early phase of the Influenza A (H1N1) pandemic in the Netherlands: results of three consecutive online surveys. BMC public health. 2011 Jan 3; 11(2):1–13. 21199571 10.1186/1471-2458-11-2 PMC3091536

[26] Stump TK, LaPergola CC, Cross NA, Else-Quest NM. The Measure of Disease-Related Stigma: Construction, validation, and application across three disease contexts. Stigma and Health. 2016 May; 1(2): 87–100.

[27] Field A. Discovering Statistics Using IBM SPSS Statistics. 5th ed. UK: SAGE; 2018.

[28] Strauss A, Corbin J. Basics of qualitative research. US: SAGE; 1990.

[29] Your research: Is it subject to the WMO or not. [Internet]. Central Committee on Research Involving Human Subjects; [Cited 2021 Apr 16]. Available from: https://english.ccmo.nl/investigators/legal-framework-for-medical-scientific-research/your-research-is-it-subject-to-the-wmo-or-not

[30] Kaiser H. F., & Rice J. Little jiffy, mark IV. Educ Psychol Meas. 1974; 34(1):111–117.

[31] Liu K, Chen Y, Lin R, Han K. Clinical features of COVID-19 in elderly patients: A comparison with young and middle-aged patients. J Infect. 2020 June; 80(6):14–18 doi: 10.1016/j.jinf.2020.03.005 32171866 PMC7102640

[32] Gedragswetenschappelijk onderzoek COVID-19: Beleving coronacrisis. [Internet]. RIVM; 2020 [Cited 2020 July 3]. Available from: https://www.rivm.nl/gedragsonderzoek/beleving-coronavirus

[33] Sharot T. The optimism bias. Curr Biol. 2011 Dec 6; 21(23):941–945. 10.1016/j.cub.2011.10.030 22153158

[34] Wolfers M, De Zwart O, Kok G. Adolescents in The Netherlands Underestimate Risk for Sexually Transmitted Infections and Deny the Need for Sexually Transmitted Infection Testing. AIDS Patient Care and STDs. 2011; 25(5)311–319 doi: 10.1089/apc.2010.0186 21542726

[35] Arnett JJ. Optimistic bias in adolescent and adult smokers and nonsmokers. Addict Behav. 2000; 25(4):625–632. doi: 10.1016/s0306-4603(99)00072-6 10972456

[36] Seale H, Heywood AE, Leask J, Steel M, Thomas S, Durrheim DN, et al. COVID-19 is rapidly changing: Examining public perceptions and behaviors in response to this evolving pandemic. PLoS one. 2020 June 23; 15(6):e0235112. doi: 10.1371/journal.pone.0235112 32574184 PMC7310732

[37] Heine SJ, Lehman DR. Cultural variation in unrealistic optimism: Does the West feel more vulnerable than the East? J Pers Soc Psychol. 1995; 68(4):595–607.

[38] Pfattheicher S, Nockur L, Böhm R, Sassenrath C, Petersen MB. The emotional path to action: empathy promotes physical distancing and wearing face masks during the COVID-19 pandemic. Psychol Sci. 2020; 31(11):1363–1373 doi: 10.1177/0956797620964422 32993455

[39] Lunn PD, Timmons S, Belton CA, Barjaková M, Julienne H, Lavin C. Motivating social distancing during the COVID-19 pandemic: an online experiment. Soc Sci Med. 2020; 265:113478 doi: 10.1016/j.socscimed.2020.113478 33162198

[40] Banker S, Park J. Evaluating prosocial COVID-19 messaging frames: Evidence from a field study on Facebook. Judgm. Decis. Mak. 2020; 12(6):1037–1043

[41] Campos-Mercade P, Meier AN, Schneider FH, Wengström E. Prosociality predicts health behaviours during the COVID-19 pandemic. Soc Sci Med. 2021; 195:104367 doi: 10.1016/j.jpubeco.2021.104367 33531719 PMC7842154

[42] Coronavirus: feiten en fabels: dit zijn antwoorden op jullie vragen. NOS [Internet]. 2020 Mar 13 [Cited 2020 June 20]; Available from: https://nos.nl/collectie/13824/artikel/2327049-coronavirus-feiten-en-fabels-dit-zijn-antwoorden-op-jullie-vragen

[43] Omgaan met de gevolgen van de coronacrisis: ik voel me alleen. [Internet]. Nederlands Jeugdinstituut; 2020 Aug 4 [Cited 2020 Aug 9]. Available from: https://www.nji.nl/nl/coronavirus/Jongeren-en-jongvolwassenen/Gezond-en-chill-voelen/Ik-voel-me-alleen

[44] Kinderen zijn de onzichtbare slachtoffers van de coronacrisis. [Internet]. Unicef; 2020 [Cited 2020 Aug 9]. Available from: https://www.unicef.nl/corona

[45] de Zwart O, Veldhuijzen IK, Richardus JH, Brug J. Monitoring of risk perceptions and correlates of precautionary behaviour related to human avian influenza during 2006–2007 in the Netherlands: results of seven consecutive surveys. BMC Infectious Diseases. 2010 May 12; 10(114):1–15. doi: 10.1186/1471-2334-10-114 20462419 PMC2885389

[46] Barattucci M, Chirico A, Kuvačic G, De Giorgio A. Rethinking the Role of Affect in Risk Judgement: What We Have Learned From COVID-19 During the First Week of Quarantine in Italy. Front Psychol. 2020; 11:554561. doi: 10.3389/fpsyg.2020.554561 33132962 PMC7565677

[47] Jolley D, Douglas KM, Skipper Y, Thomas E, Cookson D. Measuring adolescents’ beliefs in conspiracy theories: Development and validation of the Adolescent Conspiracy Beliefs Questionnaire (ACSQ). Br J Dev Psychol. 2021. 10.1111/bjdp.12368 33556990

[48] Council on Communications and Media. Media use in school-aged children and adolescents. Am Acad Pediatr. 2016; 138(5)e20162592. doi: 10.1542/peds.2016-2592 27940794

