# Supplementary material for: COVID-19 related stigma and health-protective behaviours among adolescents in the Netherlands: An explorative study
Source: PLoS One. 2021 Jun 22;16(6):e0253342. doi: 10.1371/journal.pone.0253342 (PMC8219143; doi:10.1371/journal.pone.0253342)
Supplement: S3 Appendix — (DOCX) [file pone.0253342.s003.docx]

**S3 Appendix. Legends questionnaire datasets**
This document explains the variables and the data present in the datasets used for analysis.

**Dataset: Health-Protective Behaviour questionnaire**
Progress: only 100% finished questionnaires were analysed

User language: Questionnaires were only provided in Dutch (NL)

GSLCHT: gender of the participant (1=male, 2=female)

LFTD: age of the participant (numeric answer)
SCHL: name of the school of the participant (participants could choose from a list of approached schools, we do not name the schools to protect the anonymity of our participants)

GRP: the education year of the participant (1=primary school year 7, 2= primary school year 8, 3=secondary school 1^st^ grade, 4=secondary school 2^nd^ grade, 5=secondary school 3^rd^ grade)

NV: the school level of the participant (1=secondary school VMBO, 2=secondary school HAVO, 3=secondary school VWO/gymnasium, 4=primary school, 5=secondary school HAVO/VWO, 6=other, 7=secondary school VMBO/HAVO)
CHTRGRND: ethnicity of the participant (1=autochthonous Dutch, 2=other, we deleted the text based answers of the participants detailing their ethnicity with when choosing the option “other” to protect the anonymity of the participants)
VRSKNNR: participation of the participate in the Viruskenner program (1=yes, 2=no)

The following variables are statements about COVID-19 (1=true, 2=not true)
K1: COVID-19 is contagious
K2: Someone could have COVID-19 without being ill
K3: Someone could die from COVID-19

The following variables are different diseases in the question: how serious would you rate the following diseases? (1=not serious at all, 2=not serious, 3=neither not serious nor serious, 4=serious, 5=very serious)
E1_1 Diabetes
E1_2 Common cold
E1_3 COVID-19
E1_4 Heart attack

E1_5 Food poisoning

E2 How concerned are you about COVID-19? (1=not concerned at all, 2=not concerned, 3=neither not concerned nor concerned, 4=concerned, 5=very concerned)
E3 How afraid are you for COVID-19? (1=not afraid at all, 2=not afraid, 3=neither not afraid nor afraid, 4=afraid, 5=very afraid)

The following variables are different diseases in the question: what are the odds you get the following diseases within a year? (1=very unlikely, 2=unlikely, 3=neither unlikely nor likely, 4=likely, 5=very likely)
ER1_1 Diabetes

ER1_2 Common cold
ER1_3 COVID-19
ER1_4 Heart attack
ER1_5 Food poisoning

The following variables are different diseases in the question: do you think it will help taking precautionary measures to protect yourself from the following diseases? (1=not at all, 2=a little bit, 3=quite a bit, 4=definitely)

R1_1 The flu
R1_2 COVID-19
R1_3 Common cold

The following variables are different precautionary measures in the question: Of the following precautionary measures, do you think they will help in preventing a coronavirus infection? (1=certainly not, 2=probably not, 3=not probable and not improbable, 4= probably, 5=certainly)
R2_1 Avoiding crowded areas
R2_2 Paying more attention to your hygiene
R2_3 Avoiding people that are infected with the coronavirus
R2_4 Wearing masks
R2_5 Calling a doctor for advice
R2_6 Staying home from school

The following variables are different diseases in the question: do you think you could protect yourself from the following diseases? (1=not at all, 2=a little bit, 3=quite a bit, 4=definitely)

Z1_1 The flu
Z1_2 COVID-19
Z1_3 Common cold

The following variables are different precautionary measures in the question: If the government would advise one of these following precautionary measures, would you be capable to adhere to them? (1=certainly not, 2=probably not, 3=not probable and not improbable, 4= probably, 5=certainly)

Z2_1 Avoiding crowded areas
Z2_2 Paying more attention to your hygiene
Z2_3 Avoiding people that are infected with the coronavirus
Z2_4 Wearing masks
Z2_5 Calling a doctor for advice
Z2_6 Staying home from school

The following variables are different information sources in the question: how often do you use the following information sources to get information about COVID-19? (1=not at all, not much, a little, much, very much)
I1_1 Television
I1_2 Internet websites
I1_3 Social media (e.g. Instagram, Facebook, Snapchat, Twitter)
I1_4 The newspaper
I1_5 Family (e.g. parents, grandparents, siblings)
I1_6 Friends
I1_7 The teacher at school

The following variables are different information sources in the question: how reliable do you rate the following information sources to get information about COVID-19? (1=not at all reliable, not reliable, a little reliable, reliable, very reliable)
I2_1 Television
I2_2 Internet websites
I2_3 Social media (e.g. Instagram, Facebook, Snapchat, Twitter)
I2_4 The newspaper
I2_5 Family (e.g. parents, grandparents, siblings)
I2_6 Friends
I2_7 The teacher at school

**Dataset: Measure of Disease-Related Stigma**Progress: only 100% finished questionnaires were analysed

User language: Questionnaires were only provided in Dutch (NL)

GSLCHT: gender of the participant (1=male, 2=female)

LFTD: age of the participant (numeric answer)
SCHL: name of the school of the participant (participants could choose from a list of approached schools, we do not name the schools to protect the anonymity of our participants)

GRP: the education year of the participant (1=primary school year 7, 2= primary school year 8, 3=secondary school 1^st^ grade, 4=secondary school 2^nd^ grade, 5=secondary school 3^rd^ grade)

NV: the school level of the participant (1=secondary school VMBO, 2=secondary school HAVO, 3=secondary school VWO/gymnasium, 4=primary school, 5=secondary school HAVO/VWO, 6=other, 7=secondary school VMBO/HAVO)
CHTRGRND: ethnicity of the participant (1=autochthonous Dutch, 2=other, we deleted the text based answers of the participants detailing their ethnicity with when choosing the option “other” to protect the anonymity of the participants)
VRSKNNR: participation of the participate in the Viruskenner program (1=yes, 2=no)

Vignette_group: the vignette the participant has read before answering the questions (1=high-onset controllability vignette, 2=low-onset controllability vignette, 3=unknown-onset controllability vignette)

The following variables are the questions asked after reading the randomly assigned vignette story about Lyn or Lisa in this questionnaire (1=strongly disagree, 2=disagree, 3=neither disagree nor agree, 4=agree, 5=strongly agree, 6=I don’t know).
Q_1: Lyn/Lisa could have avoided getting COVID-19
Q_2: Lyn’s/Lisa’s behaviour caused her to get COVID-19

Q_3: Lyn’s/Lisa’s actions caused her to get COVID-19

Q_4: Lyn/Lisa is responsible for her COVID-19 infection

Q_5: Lyn/Lisa is accountable for her acquisition of her COVID-19

Q_6: Lyn/Lisa could have done more to prevent her COVID-19

Q_7: Lyn’s/Lisa’s COVID-19 is the result of her own negligence

Q_8: Lyn/Lisa only has herself to blame for her COVID-19

Q_9: It is Lyn’s/Lisa’s own fault that she has COVID-19

Q_10: Lyn/Lisa did something wrong

Q_11: Lyn’s/Lisa’s COVID-19 illness is due to a mistake she made
Q_12: Lyn/Lisa could not have prevented getting COVID-19
Q_13: Lyn/Lisa had no control over the cause of COVID-19

Q_14: Lyn’s/Lisa’s behaviour is frustrating

Q_15: Lyn/Lisa makes me feel irritated

Q_16: I feel aggravated by Lyn/Lisa

Q_17: I feel sadness for Lyn/Lisa

Q_18: I feel sympathy for Lyn/Lisa

Q_19: I do not feel sorry for Lyn/Lisa

Q_20: I have no compassion for Lyn/Lisa

Q_21: I avoid sitting close to people like Lyn/Lisa

Q_22: I would not mind being in a sports team with Lyn/Lisa

Q_23: I could not become close friends with Lyn/Lisa

Q_24: I would be willing to help Lyn/Lisa with her homework via telephone call

Q_25: I would be willing to do groceries for Lyn/Lisa

Q_26: I would be willing to go to a pharmacy to get medicines for Lyn/Lisa
