# Supplementary material for: COVID-19 related stigma and health-protective behaviours among adolescents in the Netherlands: An explorative study
Source: PLoS One. 2021 Jun 22;16(6):e0253342. doi: 10.1371/journal.pone.0253342 (PMC8219143; doi:10.1371/journal.pone.0253342)
Supplement: S4 Appendix — (DOCX) [file pone.0253342.s004.docx]

**S4 Appendix. Demographics of interview participants**

| *Respondent* | *Gender* | *Age* | *School level*  *(Primary/ secondary school)* | Ethnicity |
| --- | --- | --- | --- | --- |
| 1 | Girl | 13 | Gymnasium 2 (secondary school) | Turkish Dutch |
| 2 | Girl | 11 | Group 8 (primary school) | Turkish Dutch |
| 3 | Boy | 12 | Group 8 (primary school) | Japanese Dutch |
| 4 | Girl | 16 | VWO 3 (secondary school) | Dutch |
| 5 | Girl | 14 | HAVO 3 (secondary school) | Dutch |
| 6 | Boy | 14 | VWO 3 (secondary school) | Dutch |
| 7 | Boy | 12 | MAVO/HAVO 1 (secondary school) | Dutch |
| 8 | Boy | 14 | VMBO 2 (secondary school) | Dutch |
| 9 | Girl | 14 | VWO 3 (secondary school) | Dutch |
| 10 | Girl | 13 | HAVO/VWO 1 (secondary school) | Dutch |
| 11 | Boy | 13 | Gymnasium 2 (secondary school) | Dutch |
| 12 | Boy | 16 | VWO 3 (secondary school) | Indonesian Dutch |
| 13 | Boy | 15 | VWO 3 (secondary school) | Indonesian Dutch |
| 14 | Girl | 14 | VWO 2(secondary school) | Dutch |
| 15 | Boy | 10 | Group 7 (primary school) | Dutch |
